# Combination of laboratory automation and next-generation sequencing for the diagnosis of osteoarticular infections: a technical note

**DOI:** 10.3389/fsurg.2025.1717386

**Published:** 2026-01-27

**Authors:** Sara Giordana Rimoldi, Francesco Petri, Cristina Pagani, Davide Brioschi, Matteo Passerini, Marta Stracuzzi, Andrea Gori, Alberto Dolci, Alfonso Manzotti

**Affiliations:** 1 Laboratory of Microbiology and Virology, Luigi Sacco Hospital, ASST-FBF-Sacco, Milan, Italy; 2 Department of Infectious Disease, Luigi Sacco Hospital, ASST-FBF-Sacco, Milan, Italy; 3 Department of Orthopedic and Traumatology, Luigi Sacco Hospital, ASST-FBF-Sacco, Milan, Italy; 4 Department of Pathophysiology and Transplantation, University of Milano, Milan, Italy; 5 Department of Pediatrics, Pediatric Infectious Disease Unit, Luigi Sacco Hospital, ASST-FBF-Sacco, Milan, Italy; 6 Department of Biomedical and Clinical Sciences, University of Milano, Milan, Italy; 7 Centre for Multidisciplinary Research in Health Science (MACH), University of Milan, Milan, Italy

**Keywords:** culture-negative infections, lab automation, next-generation sequencing, osteoarticular infections, pathogen identification

## Abstract

Osteoarticular infections (OAIs) present a diagnostic challenge, particularly when conventional cultures fail to identify the causative pathogen. We developed and implemented a sequential diagnostic workflow for intraoperative orthopaedic tissue samples, integrating laboratory automation (LA), broth enrichment, and next-generation sequencing (NGS). Between September 2023 and March 2025, 702 samples from 117 patients with suspected OAIs were processed. LA identified pathogens in 42% of cases. Among LA-negative patients, broth enrichment and NGS yielded additional identifications in 23% and 11.5% of cases, respectively. Our workflow achieved a diagnostic result within 10 days while improving detection rates, particularly in culture-negative cases. Our experience demonstrates that combining advanced microbiology tools is feasible in the context of suspected OAIs. A multidisciplinary evaluation of results remains essential for optimal patient management.

## 1 Introduction

Osteoarticular infections (OAIs) represent a major clinical challenge, as timely and accurate identification of the causative pathogen(s) is essential to guide targeted therapy. However, a substantial proportion of OAIs remain culture-negative, and many relevant pathogens are slow-growing, necessitating up to 14 days of incubation to maximize recovery according to some authors (1). Although the implementation of blood-culture systems has improved detection times and yields in some sample types (2), intraoperative specimens—particularly bone and cartilage fragments—are often unsuitable for this approach, leaving a diagnostic gap. In recent years, two major advancements have reshaped clinical microbiology workflows. First, laboratory automation (LA) platforms (e.g., BD Kiestra™ TLA and Copan WASPLab™) enable fully automated sample plating, incubation with continuous 24/7 digital imaging, and integrated data management, reducing manual errors, and shortening turnaround times (3, 4). Second, molecular diagnostics, notably Next-Generation Sequencing (NGS), allow culture-independent detection of microbial DNA directly from clinical specimens, enhancing sensitivity for fastidious or antibiotic-exposed organisms and potentially improving the proportion of pathogens identified in OAIs (5, 6).

Therefore, we propose a combined workflow for intraoperative orthopaedic samples (particularly, fragmented of tissue such as bone and cartilage) in our Laboratory of Microbiology, integrating LA with adjunctive NGS analysis of culture-negative specimens. This approach aims to deliver definitive microbiological results within 10 days, while preserving high diagnostic yield by applying molecular methods when conventional cultures fail to identify a pathogen.

## 2 Methods

### 2.1 Samples collection

Samples from patients with suspected OAIs were collected by the orthopedic surgery team in an aseptic environment (operating room) using an open biopsy procedure, to avoid skin contact and minimize the risk of contamination. Electrocautery was avoided during sampling in order to prevent thermal damage to tissues and potential reduction in microbial yield. Tissue and bone samples were obtained using a bone biopsy trocar system, employing a single device for each anatomical site.

Depending on the intraoperative findings, different specimen types were collected, including liquid exudate or purulent material (when present), soft tissue fragments, and cortical or cancellous bone samples. All specimens were immediately placed in sterile containers and individually labeled. For each patient, six or more samples were systematically obtained and sent to the microbiology laboratory with a unique request form to ensure traceability of both the type of material and the sampling location.

Upon receipt in the laboratory, samples were processed according to standardized microbiological protocols. Liquid samples were aliquoted and directly inoculated into culture media. Tissue and bone fragments were mechanically homogenized under aseptic conditions before culture and enrichment steps. All materials were handled in a Class II biosafety cabinet to minimize contamination risk and to preserve sample integrity prior to downstream analyses, including enrichment, culture, and NGS processing.

### 2.2 LA (copan WASPLab™)

**Figure 1:**
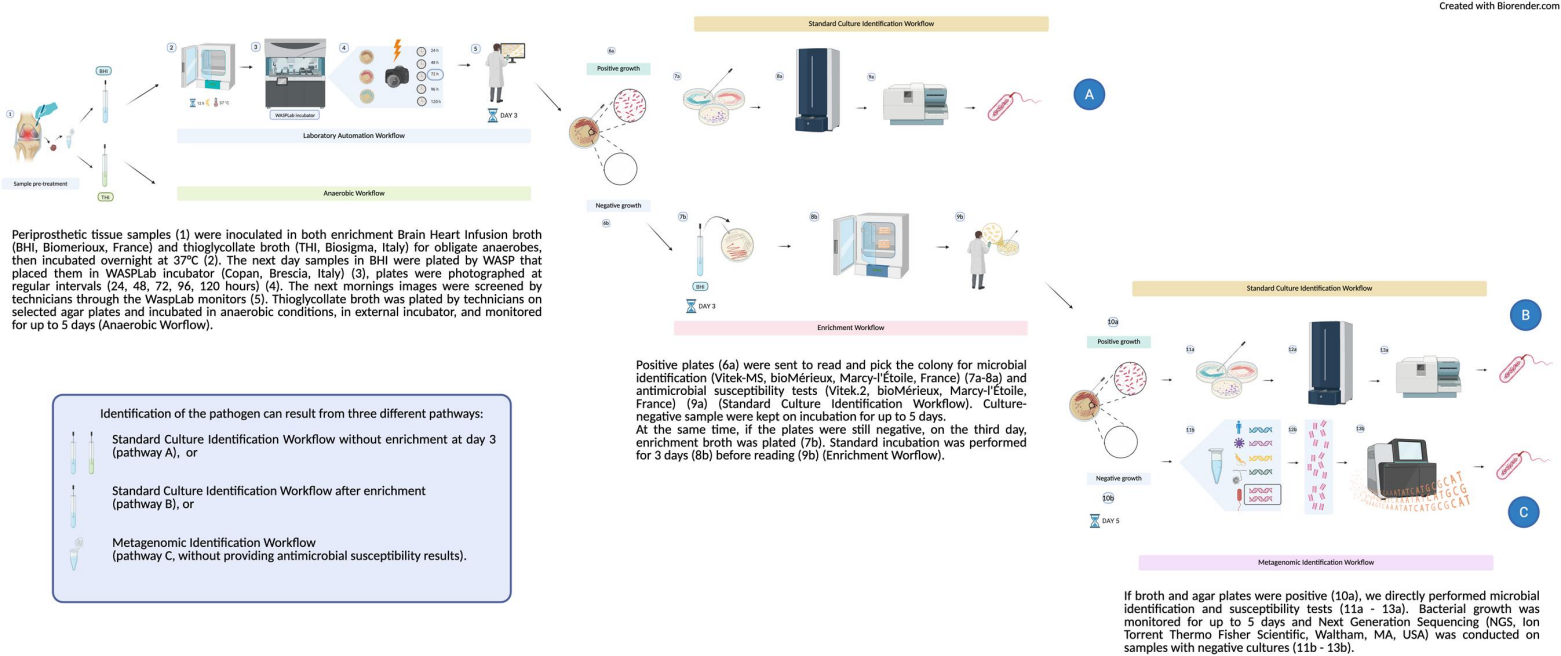
Diagnostic workflow for OAIs—including LA, broth enrichment, and NGS (see Materials and Methods)

Subculturing, aerobic and anaerobic automated and enriched workflows are depicted in Figure 1 (1–9b). Upon arrival at the laboratory, periprosthetic tissue specimens were manually inoculated in both enrichment Brain Heart Infusion broth (BHI, Biomerioux, France) and thioglycollate broth (THI, Biosigma, Italy) for obligate anaerobes, then incubated overnight at 37 °C, prior to LA processing. The next day samples in BHI were plated by WASP that placed them in WASPLab incubator (Copan, Brescia, Italy), plates were photographed at regular intervals (24, 48, 72, 96, 120 h). Each specimen was streaked in multiple plates for analysis. The subsequent mornings, images were screened by technicians through the WaspLab monitors. Thioglycollate broth was plated by technicians on selected agar plates and incubated in anaerobic conditions, in external incubator, and monitored for up to 5 days (Anaerobic Worflow). All samples are incubated in separate enrichment broths, and subsequently, each broth is individually streaked (via LA) to allow for an independent assessment of microbial growth at each anatomical site. Positive plates were sent to read and pick the colony for microbial identification (Vitek-MS, bioMérieux, Marcy-l'Étoile, France) and antimicrobial susceptibility tests (Vitek.2, bioMérieux, Marcy-l'Étoile, France) (Standard Culture Identification Workflow). Culture-negative sample were kept on incubation for up to 5 days. At the same time, if the plates were still negative, on the third day, additional enrichment broth was plated. Standard incubation was performed for 3 days before reading (Enrichment Worflow). If broth and agar plates were positive, we directly performed microbial identification and susceptibility tests. Bacterial growth was monitored for up to 5 days and NGS (Ion Torrent Thermo Fisher Scientific, Waltham, MA, USA) was conducted on samples with negative cultures. The 5-day culture duration was selected to balance diagnostic yield and turnaround time, supported by the use of broth enrichment and subsequent NGS analysis to increase the likelihood of detecting slow-growing or fastidious pathogens within a shorter timeframe. Current evidence suggests that mycobacterial cultures are not routinely indicated in low-endemic settings without specific clinical or epidemiological suspicion; such patients were excluded from our cohort (7).

### 2.3 NGS

NGS was performed on pooled samples of the same patient to examine only one sample per patient. We treated the samples according to the manufacturer's protocol (Invitrogen, Thermo Fisher Scientific, Waltham, MA, USA), and then pooled for DNA extraction. Tissue/biopsy and abscess, underwent a pre-treatment from 3 to 12 h depending on the size. DNA purification was performed using the PureLink™ Microbiome DNA Purification Kit (Invitrogen, Thermo Fisher Scientific,Waltham, MA, USA) (Figures 1, 7b).

Through the Ion 16S™ Metagenomics Kit (Ion Torrent, Thermo Fisher Scientific,Waltham, MA, USA) on Ion Chef System (Ion Torrent, Thermo Fisher Scientific,Waltham,MA, USA) and Ion GeneStudio S5 (Ion Torrent, Thermo Fisher Scientific, Waltham, MA,USA), we performed metagenomics sequencing of the 16S ribosomal RNA region (Figure 1, 11b-13b) to investigate the seven hypervariable bacterial regions (primer set V2–4–8, 3–6, and 7–9). Library preparation was performed using the Ion Plus Fragment Library Kit and theIon Xpress™ Barcode Adapters 1–16 Kit (Ion Torrent, Thermo Fisher Scientific,Waltham, MA, USA) (Figures 1, 9b). To mitigate the risk of false-positive signals due to skin flora (e.g., Coagulase-negative staphylococci) or contaminants, we applied the following interpretative criteria: only bacterial taxa present at high relative abundance were considered potentially significant; results were interpreted in conjunction with culture findings and clinical data; in doubtful cases, clinical microbiologists, orthopedic surgeons and infectious disease specialists jointly reviewed the metagenomic output. Furthermore, sequencing data were generated through a validated analytical pipeline developed with the manufacturer, and internal validation established a > 1,000-read threshold as the criterion for reliable taxonomic assignment.

NGS-derived identifications were systematically compared with culture-based results and evaluated alongside clinical presentation, thereby providing an orthogonal validation framework for confirming the microbiological significance of detected taxa.

## 3 Results

From September 2023 to March 2025, we evaluated 702 intraoperative tissue samples collected from 117 patients (6 samples per patient) with suspected or proven OAIs. The samples were processed with LA plus NGS. Forty-nine out of 117 patients resulted positive in the diagnostic of LA (Table 1). Among the 68/117 LA negative, 16 resulted positive after broth enrichment, while 6 resulted positive in the NGS analysis (Table 2).

**Table 1:** Time of bacterial growth in LA positive patients (*n* = 49).

Time of bacterial growth	No. of patients	Bacteria identified (*N*)
24 h	16	*S.aureus* (11), CoNs (2), *E. cloacae complex* (2), *E.faecalis* (1)
48 h	11	CoNs (6), *S.aureus* (4), *P.aeruginosa* (1)
72 h	9	*S.aureus* (4), CoNs (2), *E. faecalis* (2), *E.corrodens* (1)
96 h	4	CoNs (2), *E.faecalis* (1), *S.parasanguinis* (1)
120 h	9	*S.aureus* (4), CoNs (3), *Serratia sp.* (1), *E.coli* (1)

CoNs, Coagulase Negative Staphylococci.

**Table 2:** Identification of bacteria with broth enrichment and NGS in the LA negative patients (*n* = 68).

Method	Positivity (%)	Microrganism
Broth enrichment (*n* = 68)	16/68 (23%)	*S.aureus* (3), CoNs (9), *C.avidum* (1), *P.agglomerans* (1)
NGS (*n* = 52)	6/52 (11.5%)	*S.aureus* (1), CoNs (1) *C.acnes* (1), *Bacillus sp.* (1) *S.cristatus* (1), *C.tubercolostearicum* (1)

CoNs, Coagulase Negative Staphylococci.

## 4 Discussion

In this technical note, we present the diagnostic lab workflow developed at our center to improve the detection of pathogen(s) in OAIs. We employed LA as the first step; broth enrichment was performed if LA showed no culture positivity by Day 3; finally, NGS was added if both LA and broth enrichment remained negative by Day 5. Our initial clinical experience yielded encouraging results, suggesting that this sequential workflow may enhance microbiological detection in orthopaedic samples while maintaining the overall diagnostic timeframe to within 10 days.

Our proposed workflow contributes to the ongoing discussion on how to improve the diagnostic yield of OAIs, particularly in culture-negative cases. We acknowledge the crucial role of proper timing and sample collection, which has been shown to significantly reduce the proportion of culture-negative PJI cases (8). Every effort should be made, whenever the clinical scenario allows, to ensure optimal sample collection—this includes an appropriate antibiotic washout period, obtaining an adequate number of samples, proper preservation and transportation of specimens, and adherence to a rigorous laboratory workflow. However, we also recognize that in real-life clinical practice, these optimal conditions are not always met. Moreover, even when sample collection is performed under ideal conditions, a proportion of cases remain culture-negative, underscoring the need for improved diagnostic strategies. On one hand, we believe that LA enhances diagnostic performance by simplifying laboratory procedures, reducing manual errors, and shortening turnaround times. On the other hand, following the approach advocated by other research groups, we support the idea that the selective use of molecular diagnostics in culture-negative OAIs, particularly due to antibiotic treatment, can enhance diagnostic yield while minimizing unnecessary testing and reducing the risk of misinterpretation in culture-positive cases (9–11). Lastly, broth enrichment has the potential to improve sensitivity and shorten the results, as showed from similar experience (12). We found 71/117 patients with a positive results between LA, broth enrichment, and NGS. Our “large” inclusion criteria (suspected or proven OAIs) could have included also true negative samples. For future studies, we suggest a stricter inclusion criterion to improve the external generalizability of the results. Interestingly, our negative results came from three different diagnostic “methods” (LA, broth enrichment, and NGS) which hypothetically could reassure that the negativity is a true negativity.

Some challenges and limitations must be acknowledged. First, anaerobic cultures are not performed within the LA system currently in use at our center but are instead processed separately using standard manual techniques in dedicated anaerobic incubators. Second, the implementation of this workflow—particularly the NGS component—requires adequate training and highly specialized personnel, which currently limits its widespread adoption. Nevertheless, LA, while still demanding a training phase for laboratory staff, offers significant advantages in terms of process standardization and automation. Third, the diagnostic workflow described in this technical note does not include the use of blood culture bottles (2). We would like to clarify that, at our center, blood culture bottles are routinely used for the microbiological analysis of orthopaedic samples. However, the focus of this workflow is on specimens that are inherently difficult to cultivate using blood culture systems, such as bone and/or cartilage fragments. Fourth, interpretation of both broth enrichment and NGS results must also be approached with caution. A multidisciplinary team is essential to assess the clinical relevance and pathogenicity of detected organisms, especially considering the potential for environmental or handling-related contamination (13). Fifth, we would like to emphasize that this article is a technical note, with the primary objective of describing our center's experience and demonstrating the feasibility of a multimodal diagnostic approach. However, we acknowledge that, for a more rigorous evaluation of the workflow's performance—particularly to rule out an increase in false-positive rates—negative controls will be essential in future studies. Specifically, we recognize the importance of including two types of negative controls: (i) infection-negative tissue samples obtained using the same surgical procedures, to map potential procedural contaminants; and (ii) extraction-negative controls, devoid of patient material but processed identically from the moment of arrival in the laboratory, in order to detect contamination introduced by reagents and/or the laboratory environment, as recommended in the literature (14). Sixth, it is important to note that the current design of our workflow—where NGS is performed only on samples that remain culture-negative at Day 5—may limit the detection of certain pathogens, particularly in the context of polymicrobial infections. This approach, while optimized for cost-effectiveness and workflow efficiency, may indeed result in false negatives for non-dominant or fastidious organisms when a culture yields a single, cultivable species. This limitation is reflected in our relatively low proportion of polymicrobial infection detection. Running NGS concurrently with conventional methods could potentially improve sensitivity and reduce turnaround time but would significantly increase costs and require further optimization of the clinical decision-making framework. Future refinements of the workflow may consider a more selective or parallel application of NGS in culture-positive cases, particularly when clinical or intraoperative findings suggest a polymicrobial etiology. Lastly, but importantly, this article is presented as a technical note, aiming to describe our center's experience, demonstrate the feasibility of such a multimodal approach, and highlight the limitations encountered during its implementation. As such, clinical data—such as patients' baseline characteristics, antibiotic use prior to surgery, or clinical follow-up—are not included. We recognize that such data are crucial from a clinical utility perspective to demonstrate the effectiveness of our approach, and they will be incorporated in future studies.

In conclusion, we developed a sequential, feasible, and fast diagnostic workflow for OAIs that leverages multiple detection strategies—including LA, broth enrichment, and NGS—to improve pathogen identification. This approach adapts to varying diagnostic scenarios and maximizes yield within a clinically acceptable timeframe. We recommend that each center involved in the management of OAIs establish a diagnostic workflow tailored to local resources and supported by the latest scientific literature. In cases of persistent clinical suspicion despite negative cultures, in complex or recurrent infections, or among high-risk patients with prior antibiotic exposure, molecular techniques—or referral to a reference center—should be considered as valuable diagnostic options. This could optimise the use of molecular techniques, such as NGS, by ensuring they are used on patients who will benefit most. Finally, microbiological findings should always be interpreted in the context of a multidisciplinary team, involving microbiologists, infectious disease specialists, and orthopaedic surgeons, to ensure appropriate and informed clinical decision-making.

## Data Availability

The raw data supporting the conclusions of this article will be made available by the authors, without undue reservation.
